# Stress Dependence of the Small Angle Magnetization Rotation Signal in Commercial Amorphous Ribbons

**DOI:** 10.3390/ma12182908

**Published:** 2019-09-09

**Authors:** Michał Nowicki

**Affiliations:** Warsaw University of Technology, Institute of Metrology and Biomedical Engineering, 02-525 Warsaw, Poland; m.nowicki@mchtr.pw.edu.pl; Tel.: +48-690-650-386

**Keywords:** SAMR, amorphous ribbon, magnetoelastic force sensor

## Abstract

The results of the investigation on tensile stress dependence of the SAMR (small angle magnetization rotation) signal in soft magnetic amorphous ribbons are presented. Exemplary results for commercially available, negatively magnetostrictive 2705M, 2714A, and 6030D amorphous ribbons show significant stress dependence, in contrast to positively magnetostrictive 2826MB alloy. The magnetoelastic hysteresis of the obtained characteristics is compared, as well as the influence of the biasing H field and supply current variations. Based on the results, 2705M alloy with near-zero negative magnetostriction is proposed as best suited for a SAMR-based, magnetoelastic force sensor.

## 1. Introduction

Soft magnetic amorphous ribbons are widely known for their unique mechanical and magnetic properties [1,2,3,4,5,6,7]. The magnetomechanical effects play an important role in the utilization of these materials [8,9], with magnetostriction and inverse magnetomechanical (Villari) effect [10] as main examples. The magnetostriction is of great concern due to the connection between the saturation magnetostriction constant and the relative permeability of the amorphous ribbon [11]. Because the magnetostriction is undesired for ultra-soft magnetic materials, there is an ongoing effort which brings numerous magnetostriction measurement methods [12,13].

One of the simplest of these is the small angle magnetization rotation [SAMR] method, which, due to its indirect nature, can achieve significant resolution, up to 10^−9^ [14]. It is, however, mostly applicable to near-zero magnetostrictive materials, preferably with negative magnetostriction [13]. 

The SAMR method utilizes the dependence of magnetization rotation angle *θ* in the saturated sample on saturating the *H*_‖_ field value, small excitation AC field value *H*_⊥_, tensile stress *σ*, and saturation magnetostriction constant *λ_S_*. The saturating DC field *H*_‖_ is typically supplied by a long solenoid or Helmholtz coils axial with the length of the sample, while the *H*_⊥_ field is provided by a pair of coils [15], yoke [16] or even AC electric current along the sample [14]. The magnetization rotation angle is measured with the help of a measurement coil along the sample, as there is induced voltage of double the excitation *H*_⊥_ field frequency [15].

(1) $$e_{2f}=-N\cdot S\cdot \frac{d}{dt}\left(4\pi M_{S}\cos\theta \right)$$

where *N* is the number of turns of the sense coil, *S* is the cross-sectional area of the ribbon, *M_S_* is the saturation magnetization, *θ* is the angle between magnetization vector and ribbon axis. The angle *θ* can be obtained by minimization of total free energy [14].

(2) $$\theta =\frac{\mu_{0}M_{S}H_{⊥}}{\mu_{0}M_{S}H_{∥}+3\lambda_{S}\sigma }$$

From this relation, we obtain the widely known equation for *λ_S_*, given that we vary only the tensile stress *σ* and *H*_‖_ field keeping the e_2*f*_ signal constant [13].

(3) $$\lambda_{S}=\frac{-\mu_{0}M_{S}}{3}\left(\frac{\Delta H_{⊥}}{\Delta \sigma }\right).$$

The above equations were modified by later researchers to take into account angle skew, local anisotropy, etc. [15], in pursuit of 10^−9^ resolution of *λ_S_* measurement. Further works also included such aspects as the influence of torsion on saturation magnetostriction, etc. [17,18]. All of those works were focused on the measurement of saturation magnetostriction of a given material.

However, Equations (1) and (2) suggest new possible utilization of the SAMR method, which is the purpose of this paper. There is ongoing effort to develop new kinds of force sensors [19], along with magnetoelastic force sensors, play a unique role [20]. While the SAMR method was intended for research into magnetostriction behavior of soft magnetic materials, the above equations, as well as measurement practice, suggest a significant influence of tensile stresses on the magnetization rotation angle *θ*, and thus on the $e_{2f}$ signal induced in measurement coil.

It is, therefore, possible to develop a new kind of magnetoelastic force sensor, utilizing a non-balanced SAMR signal. Both above, simplified equations, as well as more sophisticated ones [14], give monotonous and unambiguous dependence of induced voltage on applied stress, which is appropriate for sensing applications. Moreover, the SAMR signal is better for materials with near-zero magnetostriction [13], which is interesting and uncommon for magnetoelastic sensor construction [21].

As the SAMR method theory relies on some simplifying assumptions, the influence of tensile stress on SAMR signal in commercial amorphous ribbons is investigated, to propose the best-suited material for new SAMR-based magnetoelastic sensor development. As can be seen, due to anelastic phenomena, magnetoelastic hysteresis [22] and apparent Villari point [23,24,25,26], obtained characteristics are far from monotonous, with distinct local maximum, nor unambiguous, due to magnetoelastic hysteresis observed in most of the magnetoelastic systems. Based on these experimental results, the most promising of the tested materials is proposed.

## 2. Materials and Methods

### 2.1. Utilized Samples

The ribbon samples used in the investigation were made from commercially available, amorphous alloys listed in Table 1. Co_70_Fe_5_Ni_2_Mo_5_B_3_Si_15_, Co_66_Fe_4_B_14_Si_15_Ni_1_, and Co_84_Fe_1.5_Mo_2_Mn_1.5_Si_7_B_2_ alloys were chosen due to their varying negative magnetostriction, and Fe_40_Ni_38_Mo_4_B_18_ due to positive magnetostriction, for comparison purposes. The ribbons were prepared by the manufacturers with standard rotating cylinder rapid quench technique [27].

### 2.2. Measurement Method

**Figure 1:**
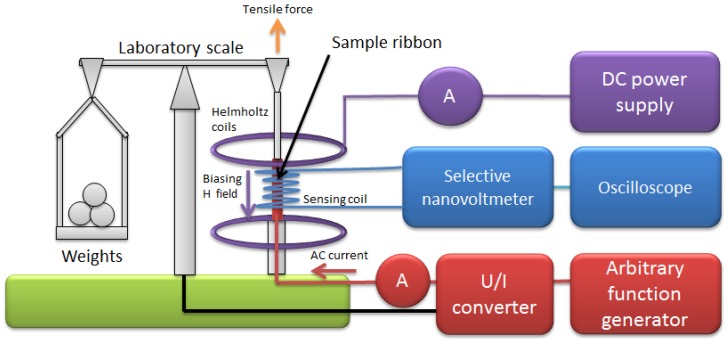
Schematic diagram of the developed small angle magnetization rotation (SAMR) system.

The measurements were carried out on a specially designed measurement system. Figure 1 presents the schematic diagram of this system.

The sample ribbons were mounted coaxially with a sensing coil and biasing Helmholtz coil in soldered mounts. The tensile stress was generated in the samples using an equal-arms laboratory scale and weights, which allows for very low values of initial stress and its precise control.

The Helmholtz coils (ESP, Warsaw, Poland) were connected to a variable bipolar DC power supply (P314, Meratronik, Warsaw, Poland) and ammeter (TH1961, Tonghui, Changzhou, China) to set a stable DC biasing/saturating field *H*_‖_. The biasing field was set to 160 A/m, which was enough to saturate the utilized high-permeability samples.

The exciting transversal field *H*_⊥_ was generated in the sample by means of an AC electrical current passed along it, with help of an arbitrary function generator (SDG1025, Siglent, Helmond, The Netherlands) connected to a voltage-current converter (RDM-2a, WUT, Warsaw, Poland) and ammeter (TH1961, Tonghui, Changzhou, China). The AC current was set to 300 mA, which is similar to common practice [14]. The frequency was 432 Hz to suppress the signal noise level.

The SAMR signal induced in the sensing coil of 1000 turns was measured with the sharp band-pass filter and amplifier (Selective Nanovoltmeter 233, Unipan, Warsaw, Poland), the output of which was fed to an oscilloscope for monitoring (model 5228, Schlumberger/Sefram, St Etienne, France).

## 3. Results and Discussion

**Figure 2:**
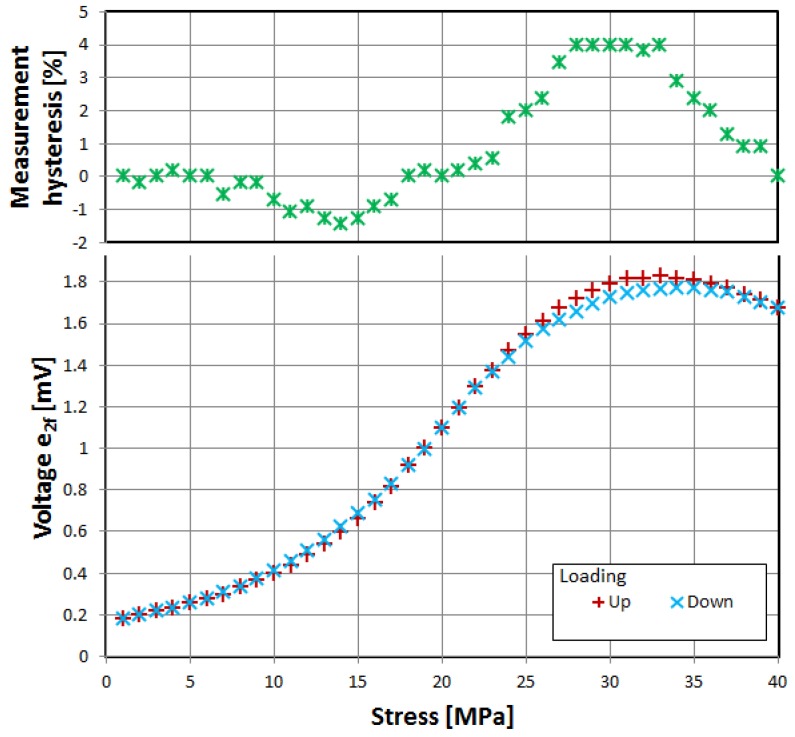
Dependence of induced e_2f_ voltage on tensile stress, alloy 2705M.

**Figure 3:**
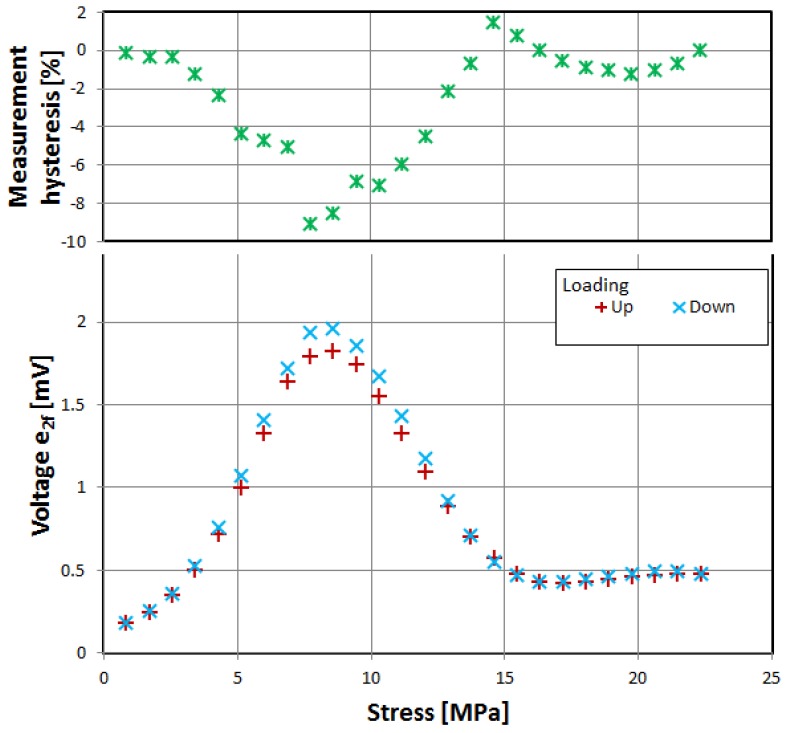
Dependence of induced e_2f_ voltage on tensile stress, alloy 2714A.

**Figure 4:**
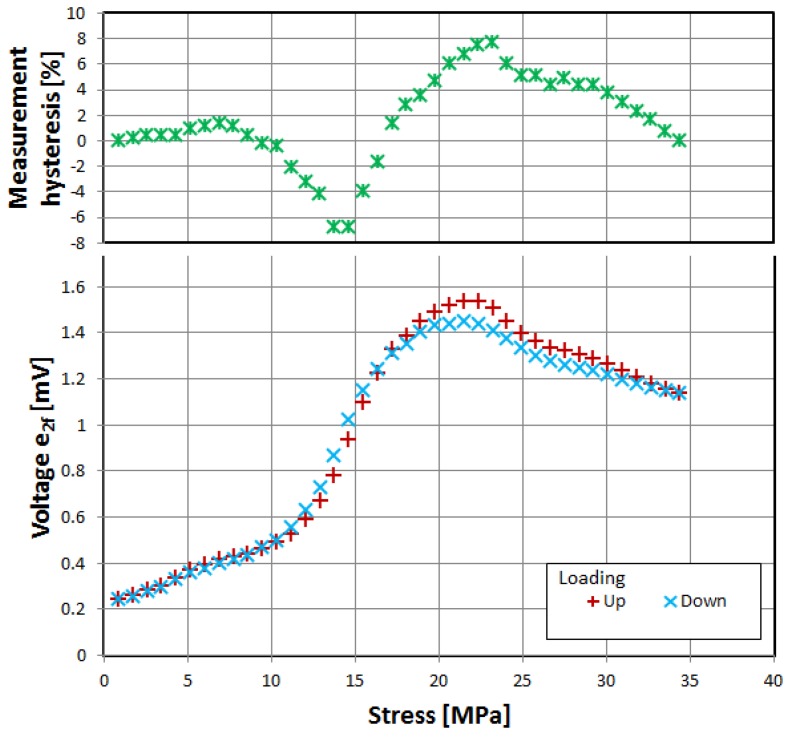
Dependence of induced e_2f_ voltage on tensile stress, alloy 6030D.

First, three of the investigated materials exhibited the significant influence of tensile stress on the magnetization rotation angle, as evidenced by characteristics of induced voltage presented in Figure 2, Figure 3 and Figure 4. The voltage measurement uncertainty was smaller than the size of points used (1% at most). The uncertainty of applied stress was less than 0.5% and was mostly due to sample cross-section uncertainty.

The samples were gradually loaded with increasing stress up to full load, and then stress gradually decreased. Thus, measurement hysteresis was investigated, which is, for the most part, due to magnetoelastic hysteresis of the sample’s material, and is one of the ‘bottlenecks’ of accuracy for magnetoelastic-based force sensors, and needs additional nontrivial compensation [30,31].

All three of investigated negative-magnetostrictive amorphous alloys exhibited similar behavior, with a distinct maximum for a particular value of stress, reminiscent of the Villari reversal point of typical magnetoelastic characteristics [23]. From Equations (1) and (2), it is evident that the angle *θ*, and thus the obtained signal e_2*f*_, depends on the stress-induced anisotropy of the material. However, for a given set of material constants *M_S_* and *λ_S_*, the stress-induced voltage should rise monotonically. The local maximum and gradual fall of the induced signal can be explained by the stress-induced change in value and sign of the saturation magnetostriction *λ_S_*. The same effect was proposed as responsible for the Villari reversal point in standard magnetoelastic characteristics [26]. 

The measurement hysteresis was most profound near the local maximum, reaching as high as −9% for 2714A alloy. 

Alloy 2705M exhibited both the widest range of stress before reaching local maximum and lowest values of measurement hysteresis, giving about 1% uncertainty in the 0 to 20 MPa range.

**Figure 5:**
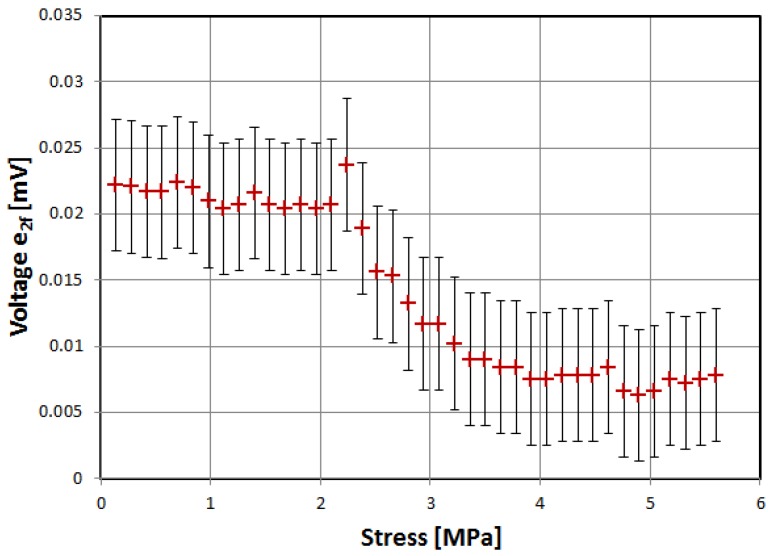
Dependence of induced e_2f_ voltage on tensile stress, alloy 2826MB. Due to the low-level signal, and thus high uncertainty, measurement hysteresis was not recorded.

The characteristic of positively magnetostrictive 2836MB alloy is different (Figure 5)—measurable changes took place for much lower stress values, and there was no distinct local maximum (which would correspond to standard magnetoelastic characteristics [32]). The overall signal amplitude was nearly 100 times smaller than for the negative-magnetostriction samples, however, which is one of the reasons why standard SAMR determination of *λ_S_* is best suited to negative-magnetostrictive materials.

**Figure 6:**
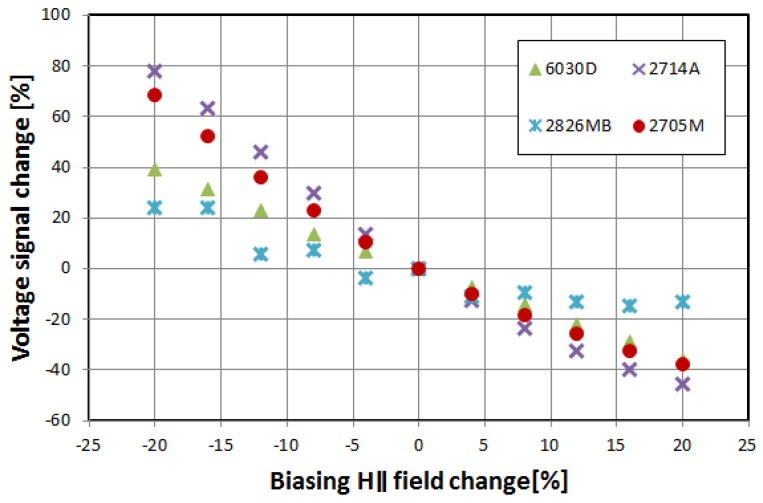
Relative change of the induced e_2f_ voltage in the function of biasing *H*_‖_ field change.

**Figure 7:**
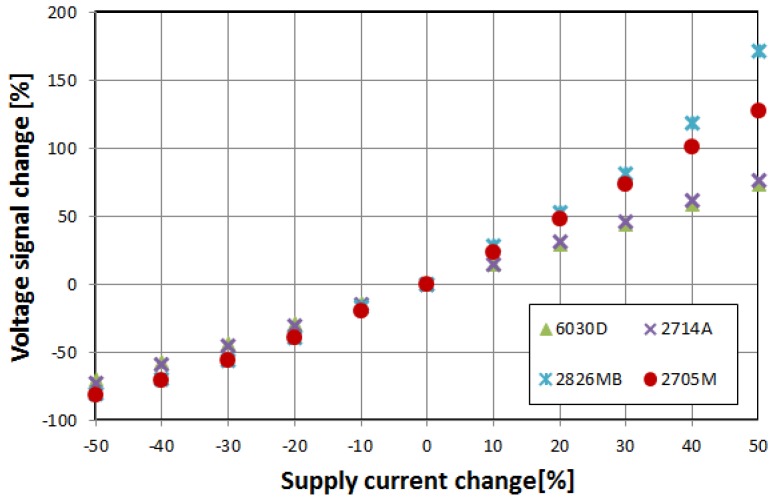
Relative change of the induced e_2f_ voltage in the function of supply current change, and thus proportional exciting *H*_⊥_ field change.

Figure 6 and Figure 7 show the relative change of induced voltage e_2*f*_, and thus magnetization rotation angle *θ*, on the parallel biasing *H*_‖_ field and, indirectly, transversal *H*_⊥_ field variations. The characteristics are surprisingly similar for all of the tested alloys, and correspond with Equations (1) and (2)—the induced voltage rises for rising *H*_⊥_, and falls for rising *H*_‖_.

The sensitivity to the external magnetic field is similar to the orthogonal fluxgates operational principle [33]. It is interesting to note that for the samples of the same size and shape, the sensitivity is almost the same, regardless of sample relative permeability. It is mostly due to assumed technical magnetic saturation of the material—in Equations (1) and (2) the relative permeability of the samples is absent. 

## 4. Conclusions

The measurement stand capable of measurements of tensile stress influence on the small angle magnetization rotation signal was presented. The e_2*f*_(*σ*) characteristics of four commercial amorphous ribbons were investigated, including the bane of magnetoelastic technology, which is magnetoelastic hysteresis. The negative-magnetostrictive ribbons exhibited similar characteristics, with distinct local maximum similar to the Villari reversal point. The measurement hysteresis was unacceptably high for 2714A and 6030D alloys. The positive-magnetostrictive alloy 2826MB is unsuitable for further investigation due to the very low-level signal obtained. Thus, opposite to typical *B*(*σ*) or *µ*(*σ*) magnetoelastic force sensors development [34,35,36], near-zero, negative magnetostriction 2705M alloy is proposed as the most promising for unbalanced SAMR-based magnetoelastic force sensor construction.

The responsivity frequency bandwidth of the proposed sensor would be limited by the supply current frequency. The measurement range is limited by the Villari reversal point. Thus reproducible results are to be expected in the 0 to 25 MPa range of the core stress. For the presented case, it translates to approximately the 0 to 50 N force range. This range is, however, scalable with the core cross-section.

## Figures and Tables

**Table 1:** Essential parameters of investigated samples, according to the manufacturer’s [28,29].

Manufacturer and Trade Name	Chemical Composition	Thick-Ness (µm)	Maximal Permeability in as-Cast State μ	Magneto-Striction in Saturation *λ_s_* (m/m)	Saturation Induction *B_s_* (T)	Coercivity H_c_ (A/m)
Metglas 2705M	Co_70_Fe_5_Ni_2_Mo_5_B_3_Si_15_	22	290,000	<0.5 (−1.36) ^1^	0.77	0.95
Metglas 2714A	Co_66_Fe_4_B_14_Si_15_Ni_1_	15	80,000	<0.5 (−3.75) ^1^	0.57	8
Vacuum-schmelze 6030 D30	Co_84_Fe_1.5_Mo_2_Mn_1.5_Si_7_B_2_	21	450,000	−11.8 (−10.2) ^1^	0.82	2.03
Metglas 2826MB	Fe_40_Ni_38_Mo_4_B_18_	29	>50,000	12 (N/A) ^1^	0.88	3.08

^1^ results in brackets obtained with the standard SAMR method on the presented test stand. For 2826MB SAMR signal was too low to perform *λ_s_* measurement.
